# Incidence and Risk Factors for Infections Requiring Hospitalization, Including Pneumocystis Pneumonia, in Japanese Patients with Rheumatoid Arthritis

**DOI:** 10.1155/2017/6730812

**Published:** 2017-10-18

**Authors:** Atsushi Hashimoto, Shiori Suto, Kouichiro Horie, Hidefumi Fukuda, Shinichi Nogi, Kanako Iwata, Hirotaka Tsuno, Hideki Ogihara, Misato Kawakami, Akiko Komiya, Hiroshi Furukawa, Toshihiro Matsui, Shigeto Tohma

**Affiliations:** ^1^Department of Rheumatology, National Hospital Organization Sagamihara National Hospital, Sagamihara, Japan; ^2^Department of Rheumatology, Clinical Research Center for Allergy and Rheumatology, National Hospital Organization Sagamihara National Hospital, Sagamihara, Japan; ^3^Department of Clinical Laboratory, National Hospital Organization Sagamihara National Hospital, Sagamihara, Japan; ^4^Molecular and Genetic Epidemiology Laboratory, Faculty of Medicine, University of Tsukuba, Tsukuba, Japan

## Abstract

**Objective:**

Rheumatoid arthritis (RA) may be complicated by different infections, but risk factors for these are not fully elucidated. Here, we assessed the incidence of and risk factors for infections requiring hospitalization (IRH) including pneumocystis pneumonia (PCP) in patients with RA.

**Methods:**

We retrospectively surveyed all RA patients treated at our hospital from 2009 to 2013, for whom data were available on demographic features, medications, comorbidities, and severity of RA. Multivariate logistic regression analysis was applied to calculate adjusted odds ratios (ORs) for factors associated with the occurrence of IRH.

**Results:**

In a total of 9210 patient-years (2688 patients), there were 373 IRH (3.7/100 patient-years). Respiratory tract infections were most frequent (*n* = 154, and additionally 16 PCP), followed by urinary tract infections (*n* = 50). Significant factors for PCP included higher age (≥70 years; OR 3.5), male sex (6.6), underlying lung disease (3.0), use of corticosteroids (4.8), and use of biologics (5.4). Use of methotrexate (5.7) was positively associated with PCP but negatively with total infections (0.7). Additionally, functional disorders and higher RA disease activity were also related to total infections.

**Conclusions:**

Risk factors for infection should be taken into account when deciding treatment for the individual RA patient.

## 1. Introduction

Biological and other recently developed agents for treating rheumatoid arthritis (RA) now facilitate control of disease activity in many patients. Recent therapeutic recommendations include the use of these drugs, despite the fact that such potent new therapies, like the corticosteroids used for many years, can cause serious infection by virtue of their immunosuppressive activity. Thus, infection rates in RA patients are reported to be almost twice those of the general population [1] and infectious pneumonia is a common cause of death in RA [2, 3]. On the other hand, RA itself is generally not lethal, except under certain conditions such as aggravated lung involvement. It is therefore important to distinguish iatrogenic effects from disease pathology. In particular, pneumocystis pneumonia (PCP) more commonly seen in AIDS is a life-threatening infection even in human immunodeficiency virus- (HIV-) negative patients (including RA patients) with a mortality rate higher than in HIV-positive patients [4, 5]. Potent therapies for RA can cause lethal infections, especially in elderly patients or those with comorbidities. Clinicians should ideally be able to estimate the risk and benefit of a particular treatment and match this to an optimal individualized therapy for each RA patient. To this end, we investigated the incidence of infections requiring hospitalization (IRH), including PCP, and factors related to their occurrence in a cohort of RA patients.

## 2. Materials and Methods

### 2.1. Patients

Every patient with RA who visited Sagamihara National Hospital from April 2009 to March 2013 was enrolled in this study and their clinical information was retrospectively obtained from their medical records. All the subjects met the standard diagnostic criteria for RA [6, 7]. The data on all patients have been obtained every year by means of an open prospective cohort registry, which meant that not only fixed patients were continuously followed and registered, but the accumulation of patient data collected every year was analyzed using patient-year method. This study was approved by Sagamihara National Hospital Research Ethics Committee. Patients receiving JAK inhibitors (e.g., tofacitinib) or any biologics other than infliximab, etanercept, adalimumab, golimumab, tocilizumab, or abatacept were excluded because they were too few in number for meaningful stratification. Patients on clinical trial or taking over the recommended dose of biologics were also excluded.

### 2.2. Clinical Information

In multivariate analysis of risk factors for each IRH, the following parameters were assessed: age, sex, RA disease duration, and RA disease activity score in 28 joints with erythrocyte sedimentation rate (DAS28-ESR), Steinbrocker's stage score, global functional status, underlying lung disease, renal function, use of nonsteroidal anti-inflammatory drugs (NSAIDs), corticosteroids, methotrexate (MTX), bucillamine, or salazosulfapyridine, and the above-mentioned biologics and immunosuppressants (tacrolimus, azathioprine, mizoribine, cyclophosphamide, or cyclosporine). In Japan, tacrolimus is the most common immunosuppressant used to treat RA; the biologics included all that had been approved for RA by 2012. The stage of articular destruction was classified from radiographs of the hands based on Steinbrocker's classification (Stage) [8]. Global functional status (Class) was based on classification by the American College of Rheumatology 1991 revised criteria [9]. Estimated glomerular filtration rate (eGFR) as a measure of renal function was calculated from the serum creatinine level using equations developed by the Japanese Society of Nephrology [10]. Patients with an eGFR < 60 ml/min/1.73 m^2^ were regarded as having renal dysfunction. Underlying lung disease was identified by computed tomography (CT) of the lung during the study period; patients who did not undergo CT were regarded as being free of lung disease. CT of the lung was performed in 35.8% of the patient cohort. Annual clinical information was averaged from regular visits over the year, whereas IRH were recognized at hospitalization or for other events including infection occurring outside of regular visits.

Diagnosis of individual infections was based on clinical and laboratory findings. PCP was diagnosed when a patient with fever and/or nonproductive cough had progressive hypoxemia, ground glass opacity of the lung detected by CT, and positivity for* Pneumocystis jirovecii* in the sputum by PCR, or elevated serum (1-3)-*β*-D-Glucan, as described in previous studies [11, 12].

### 2.3. Statistical Analysis

Between-groups comparisons for univariate analysis were conducted using Student's *t*-test or Wilcoxon rank sum test for continuous variables or Fisher's exact test for categorical variables. *p* values < 0.05 were considered significant. Associations of relevant factors with each particular infection were estimated using multivariate analysis with multiple logistic regression. To select variables for multivariate analysis, stepwise regression was applied and only significant variables were employed for the analysis. The Wald test was used to assess the significance of each factor. Threshold values of variables such as age and DAS28-ESR were calculated by univariate analysis using receiver operating characteristic (ROC) curves. For infections with which the use of corticosteroids was significantly associated in multivariate analysis, threshold doses of corticosteroids (prednisolone equivalents) were calculated in a similar manner.

## 3. Results

### 3.1. Patient Demographics

Table 1 shows the characteristics of patients with or without infection given as patient-years. In the whole cohort of 2688 patients (9210 patient-years), 274 patients suffered 373 IRH. Fifty-four had multiple such infections in different years. The overall rate of IRH was 3.7 per 100 patient-years; 11.5% had renal dysfunction (eGFR < 60 ml/min/1.73 m^2^) with 3.3% having an eGFR < 45 ml/min/1.73 m^2^. Relative to patients without infection, factors identified by univariate analysis as potentially associated with IRH were female sex, higher age, longer RA disease duration, higher rheumatoid factor, having renal dysfunction, having lung disease, higher RA disease activity, higher stage of RA, lower functional status, and having been treated with corticosteroids, biologics, or immunosuppressants (with the exception of MTX).

### 3.2. Sites and Types of Infections

**Figure 1:**
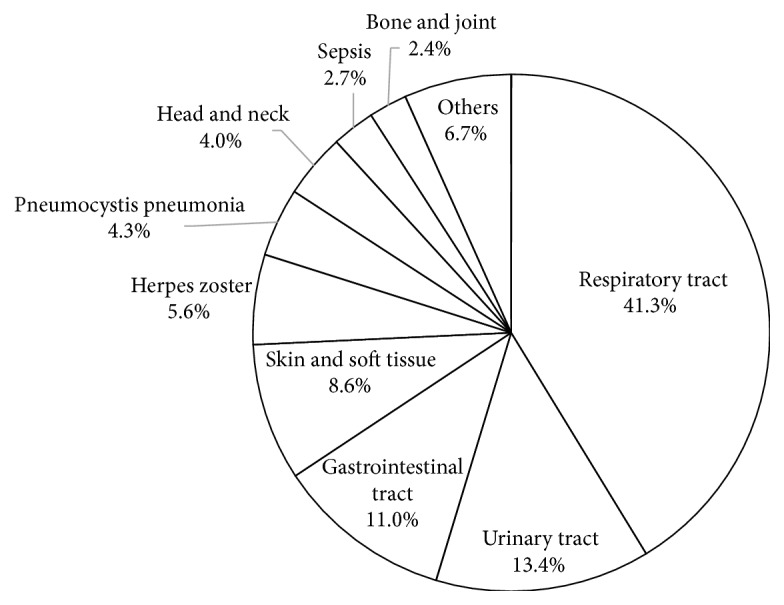
Sites and types of infections. Gastrointestinal tract includes biliary tract.

Figure 1 illustrates the sites and types of IRH present in these patients. Simultaneous multiple infections are recorded as separate events. PCP and tuberculosis are listed separately from respiratory tract infections, and herpes zoster (HZ) is listed separately from skin and soft tissue infections in the figure. “Others” include 8 other viral infections and 5 tuberculosis patients. The respiratory tract was the most frequent site of infection (*n* = 154, 41.3%) followed by the urinary tract (*n* = 50, 13.4%), gastrointestinal tract including biliary tract (*n* = 41, 11.0%), skin and soft tissue (*n* = 32, 8.6%), HZ (*n* = 21, 5.6%), and PCP (*n* = 16, 4.3%). Incidence rates of all infections and of the common infections are summarized in Table 2.

Sixteen patients suffered from PCP; none had received any prophylaxis. All had been treated with either corticosteroids (*n* = 14) or MTX (*n* = 13). Biologics had been administered to 6 patients (one each receiving infliximab, golimumab, or abatacept and three with adalimumab). Duration of treatment with these biologics before the onset of PCP ranged from 2 to 8 months (median 3 months).

### 3.3. Risk Factors

Table 2 shows the significant risk factors and odds ratios (ORs) with 95% confidence intervals (CIs) for individual infections estimated using multivariate analysis. Threshold values of age (70 years) and DAS28-ESR (3.8) were calculated by univariate analysis using ROC curves. Threshold doses of corticosteroids for individual infections were similarly estimated. Significant risk factors for any infection were age ≥ 70 years, male sex, Class III or IV, having underlying lung disease, DAS28-ESR ≥ 3.8, use of corticosteroids, and use of biologics. Use of corticosteroids was a significant risk factor for any infection and for common infections, whereas use of MTX was significantly associated with a reduced risk for most infections except for PCP. Furthermore, no significant difference was found in background factors when comparing 54 patients with multiple infections with 220 patients with only one infection.

Higher RA disease activity (DAS28-ESR ≥ 3.8) was a risk factor for any infection, as was renal dysfunction (eGFR < 60 ml/min/1.73 m^2^) for urinary tract infection. The only significant risk factor for HZ was use of corticosteroids. We also analyzed each of the 6 biologics separately or as a group of tumor necrosis factor- (TNF-) directed versus not TNF-directed biologics as a variable for any infections but found no significant correlations (data not shown).

Significant risk factors for PCP were age ≥ 70 years, male sex, having underlying lung disease, use of corticosteroids, use of biologics, and use of MTX. Of 16 patients with PCP, all had three or more risk factors and 11 patients (68.8%) had received corticosteroids and MTX simultaneously. Classifying every patient-year with ≥3 risk factors including corticosteroids and MTX as having a high risk for PCP, the sensitivity, specificity, and positive and negative predictive values for PCP were estimated as 68.8%, 81.7%, 0.7%, and 99.9%, respectively.

## 4. Discussion

Risk factors for any type of infection in RA patients, especially those associated with the use of biologics, have been well-investigated, but few studies have described the individual incidence of the different common infections and their related susceptibility factors. Accordingly, here we identified the most frequent sites for and types of IRH and the associated risk factors for these in a cohort of RA patients. It is important to note that the characteristics of the RA patients in our cohort correspond well to those described for the ten-year cumulative dataset from the largest nationwide Japanese multicenter cohort of RA patients (NinJa, the National Database of Rheumatic Disease by iR-net in Japan registry) [13], implying that our cohort is representative of the overall situation of RA patients in Japan. In this cohort, the rate of hospitalization for infections (3.7/100 patient-years) was fairly similar to previous reports (approximately 1 to 10 per 100 patient-years) with several factors accounting for variability, such as heterogeneity among the cohorts and decisions for hospitalization [1, 14–20]. Our results confirmed established data that the most frequent infection site in RA patients is the respiratory tract, accounting for approximately one-third to one-half of all infections [1, 14, 16, 18, 19, 21, 22]. Similarly, the next most frequent infection sites were the urinary tract, skin and soft tissue (including HZ), and the gastrointestinal tract [1, 14, 19, 22].

Numerous studies have noted different risk factors for total serious infections in RA patients, attributed to three major factors: immunological dysfunction resulting from RA itself, organ involvement of RA and other comorbidities, and immunosuppressive effects of medication [23]. These are, however, related to each other and cannot be clearly separated. Factors other than medications are also important because frequent infections in RA patients have been a problem since the presteroid era [24] and higher RA disease activity assessed as increasing DAS28 was reported to be associated with IRH [15]. Thus, Crowson et al. performed detailed risk assessment scoring to predict serious infection and reported that significant risk factors were higher age, previous serious infection, use of corticosteroids, elevated erythrocyte sedimentation rate, extra-articular RA manifestations, and comorbidities including coronary heart disease, heart failure, peripheral vascular disease, chronic lung disease, diabetes mellitus, or alcoholism [17].

As with the present study, earlier reports demonstrated a significant risk of infection when using corticosteroids [14, 17, 21, 23]. In the present study, we calculated threshold doses of corticosteroids for individual infections using univariate analysis. The threshold dose of corticosteroids for PCP was higher than for the other infections (Table 2), suggesting that PCP develops only under highly immunosuppressed states in RA patients.

Biologics are assumed to be contributing to the increased susceptibility to infection in RA and numerous studies have addressed this issue. A recent meta-analysis of 106 randomized trials found that use of standard-dose biologics was associated with a slight but significantly increased risk for serious infections (OR 1.31, 95% CI 1.09–1.58) relative to traditional disease-modifying antirheumatic drugs (DMARDs) [20]. That OR is similar to our findings. The crucial factor here may be the time after initiation of treatment with a biological agent, with meta-analyses showing a significantly higher risk of opportunistic infections in short-term (<6 months) than in long-term studies [25], as well as a time-dependent decreasing risk of serious infection from the use of anti-TNF biologics [26]. The increased risk of infection when using biologics seems to be absent after a year has passed.

Whether MTX contributes to increased susceptibility to infection is still controversial. Some studies found an increased risk [21, 27], while others did not [15, 28, 29], or even reported a reduced risk as in our own results [14, 30]. Given that high RA disease activity predisposes to infection, both anti-rheumatic and immunosuppressive effects of medication need to be assessed. Immunosuppressive effects of MTX, if any, will likely be offset by its activity in improving immunological dysfunction caused by RA [31]. Of note, in the present study MTX use was associated with a reduced risk for most infections but an increased risk solely for PCP. Previous studies have also indicated associations between MTX use and PCP [32, 33]; however, the detailed mechanism of the PCP-specific risk of MTX use is unclear. MTX-induced total lymphopenia or depletion of CD4+ cells may contribute to some degree, but the majority of studies showed no significant differences in peripheral blood total lymphocyte counts between RA patients with or without PCP [5, 32–34]. In contrast to HIV-positive patients whose established risk for PCP is a CD4+ cell count < 200 cells/*μ*L, RA patients with CD4+ cells > 200/*μ*L often suffer from PCP [5, 32]. It is proposed that altered lymphocyte subsets other than general depletion of CD4+ cells by MTX [35] as well as by immunosuppressive therapies [36] may contribute to the development of PCP.

Postmarketing surveillance (PMS) reports in Japan revealed that the incidence of PCP in RA patients treated with biologics or iguratimod was approximately 0.1 to 0.3% at 24 weeks or 6 months after the initiation of treatment, respectively [37, 38]. Incidence estimated from the present study (0.2/100 patient-years) is in line with these results. Interestingly, the incidence of PCP reported from studies in Western countries is less than in Japan. An investigation of two US population-based hospitalization databases of RA patients from 1996 to 2007 indicated an incidence of PCP ranging from 0.6 to 4.0/100,000 patient-years without any changes even after infliximab and etanercept began to be commonly used for RA [39]. In the UK, data from the British Society for Rheumatology Biologics Register for RA were analyzed regarding incidence of PCP, which was reported to range from 1.1 (with traditional synthetic DMARDs) to 2.0 (with anti-TNF biologics) per 10,000 patient-years [40]. As mentioned above, RA patients probably have the highest risk of infections early after the initiation of biologics. Indeed, the mean duration of administration of biologics before PCP onset was 4 months in the present study and 9 weeks to 7.2 months in previous studies [12, 33, 41]. Although the incidence derived from PMS in Japan was for the period early after the initiation of biologics or iguratimod, it is possible that PCP is 10–100 times more frequent in Japan than in the US or Europe.

In RA patients, established risk factors for PCP are higher age, use of corticosteroids, and underlying lung disease [12, 34, 42]. Higher age is a common contributor to PCP as concluded by previous studies of RA using multivariate analysis [12, 33, 42]. In general, higher age [43, 44], use of corticosteroids [45, 46], and chronic lung disease [47, 48] were associated with colonization by* P. jirovecii*, which is found in up to 55% of patients with chronic obstructive pulmonary disease (COPD) [48] and 37.8% of patients with idiopathic interstitial pneumonia [47]. Mori et al. performed molecular testing for* P. jirovecii* on sputum or bronchoalveolar lavage fluids of 82 patients with RA. Nine (10.9%) asymptomatic carriers were identified, all of whom had received MTX [49]. Thus, MTX may facilitate colonization of* P. jirovecii* and be a risk factor for PCP. Additionally, we identified male sex, use of biologics, and use of MTX as risk factors, which might be useful for considering prophylaxis for PCP.

In contrast to our results, Kourbeti et al. failed to show a significant association of the use of biologics with PCP in a meta-analysis (OR 1.77, 95% CI 0.42–7.47) [25]; however, this does not necessarily imply a lack of association for several reasons. In studies in Western countries, the number of patients with PCP may be too low to be powered to detect any significant risk with biologics. Presumably, biologics confer a high risk for PCP only shortly after treatment initiation. Among studies comparing the incidence of PCP between RA patients with or without biologics, most of the latter received MTX, which might itself also predispose to PCP.

In addition to biases brought about by a retrospective single-center study, the present study has some other limitations. Information about smoking status, comorbidities such as diabetes mellitus and cardiac disorders, and history of previous infections was not available. To assess detailed risks of using biologics on infection, the history of their use, duration of administration, and dose should be included in the analysis. DAS28-ESR at only one time point as considered here (at one year) might be insufficient to fully evaluate associations between RA disease activity and infection. Sometimes PCP is hard to be distinguished from MTX or RA induced interstitial pneumonia, which could influence the risk estimation for PCP. Our study could not reveal any general features of HZ in RA patients because we assessed only hospitalized patients with HZ.

In conclusion, the present study documents the incidence of and risk factors for individual infections in a cohort of RA patients. The most frequent site of infection was the respiratory tract, while PCP was not rare in patients without prophylaxis. For both PCP and all infections, risk factors included higher age, male sex, underlying lung disease, and use of corticosteroids. Use of MTX was related only to PCP and inversely to total and common infections. Relationships between risks of infection, medication, and RA disease activity are not easy to distinguish but need to be estimated in order to provide the most appropriate treatment for infection control in each individual RA patient.

## Figures and Tables

**Table 1:** Characteristics of patients with or without infections.

	Without infection (2414 pts)	With infection (274 pts)	*p* value
Female	83.9%	73.7%	<0.0001
Age, years, mean (SD)	63.7 (12.3)	70.4 (9.9)	<0.0001
Over 70 years of age	34.4%	60.2%	<0.0001
Disease duration of RA, years, median (IQR)	12 (5–22)	16 (9–26)	<0.0001
Rheumatoid factor, mean, U/ml	142.2	240.1	<0.0001
eGFR, mL/min/1.73 m^2^, median (IQR)	80 (70–95)	75 (60–95.5)	0.002
eGFR < 60 mL/min/1.73 m^2^	11.1%	22.6%	<0.0001
Underlying lung disease	14.9%	50.0%	<0.0001
DAS28-ESR, mean ± SD	3.1 ± 1.1	3.7 ± 1.2	<0.0001
Stage III or IV	48.5%	70.1%	<0.0001
Class III or IV	14.6%	47.8%	<0.0001
Treated with corticosteroids (median dose, mg/day)	51.8% (3.75)	85.4% (5)	<0.0001
Treated with MTX (median dose, mg/week)	59.6% (8)	40.4% (8)	<0.0001
Treated with biologics	13.0%	18.1%	0.009
Etanercept	40.9%	37.7%	
Tocilizumab	25.5%	24.6%	
Adalimumab	13.9%	13.1%	
Infliximab	9.3%	6.6%	
Abatacept	6.3%	9.8%	
Golimumab	4.2%	8.2%	
Treated with immunosuppressants	7.9%	13.2%	0.001

eGFR, estimated glomerular filtration rate; DAS28-ESR, disease activity score in 28 joints with erythrocyte sedimentation rate; Stage, Steinbrocker's radiographic stage score; Class, global functional status; NSAIDs, nonsteroidal anti-inflammatory drugs; MTX, methotrexate. Dose of corticosteroids is expressed in prednisolone equivalents. Patients who did not receive corticosteroids or MTX were excluded for calculating median doses.

**Table 2:** Significant risk factors and the odds ratios for each infection.

Total (*n* = 342, 3.7/100 PY)	OR (95% CI)	*p* value
Age 70 and older	1.5 (1.2–2.0)	0.002
Male	1.6 (1.1–2.2)	0.004
Class III or IV	2.2 (1.7–3.0)	<0.0001
Underlying lung disease	3.1 (2.3–4.2)	<0.0001
DAS28-ESR ≥ 3.8	1.7 (1.3–2.3)	0.0001
Corticosteroids	3.0 (2.1–4.4)	<0.0001 ^*∗*^2.00 mg/day
Biologics	1.4 (1.0–2.0)	0.033
Methotrexate	0.7 (0.6–1.0)	0.034

Respiratory tract (*n* = 154, 1.7/100 PY)	OR (95% CI)	*p* value

Age 70 and older	1.5 (1.0–2.1)	0.031
Male	2.5 (1.7–3.6)	<0.0001
Class III or IV	2.6 (1.8–3.7)	<0.0001
Underlying lung disease	5.1 (3.5–7.4)	<0.0001
Corticosteroids	2.5 (1.6–4.0)	0.0001 ^*∗*^2.50 mg/day
Methotrexate	0.7 (0.5–1.0)	0.049

Urinary tract (*n* = 50, 0.5/100 PY)	OR (95% CI)	*p* value

Female	3.9 (1.2–23.9)	0.062
Class III or IV	7.6 (4.0–15.0)	<0.0001
eGFR < 60 ml/min/1.73 m^2^	2.2 (1.2–4.1)	0.013
Corticosteroids	3.8 (1.6–11.2)	0.006 ^*∗*^1.85 mg/day
Methotrexate	0.5 (0.3–0.9)	0.028

Gastrointestinal tract (*n* = 41, 0.4/100 PY)	OR (95% CI)	*p* value

Age 70 and older	4.0 (2.0–9.2)	0.0004
Class III or IV	3.4 (1.7–6.8)	0.0004
Corticosteroids	4.5 (1.7–15.4)	0.005 ^*∗*^1.85 mg/day

Skin and soft tissue (*n* = 32, 0.3/100 PY)	OR (95% CI)	*p* value

Class III or IV	4.1 (1.9–8.8)	0.0002
Corticosteroids	3.0 (1.2–9.0)	0.033 ^*∗*^1.75 mg/day
Methotrexate	0.4 (0.2–0.9)	0.025

Herpes zoster (*n* = 21, 0.2/100 PY)	OR (95% CI)	*p* value

Corticosteroids	5.3 (1.8–22.8)	0.007 ^*∗*^3.75 mg/day

PCP (*n* = 16, 0.2/100 PY)	OR (95% CI)	*p* value

Age 70 and older	3.5 (1.2–10.8)	0.025
Male	6.6 (2.4–19.0)	0.0003
Underlying lung disease	3.0 (1.0–8.6)	0.038
Corticosteroids	4.8 (1.3–31.0)	0.039 ^*∗*^5.00 mg/day
Biologics	5.4 (1.7–15.7)	0.002
Methotrexate	5.7 (1.8–25.9)	0.008

PY, patient-years; OR, odds ratio; CI, confidence interval; Class, global functional status; PCP, Pneumocystis pneumonia. ^*∗*^Threshold dose of corticosteroids (prednisolone equivalents).

## References

[1] Doran M. F., Crowson C. S., Pond G. R., O'Fallon W. M., Gabriel S. E. (2002). Frequency of infection in patients with rheumatoid arthritis compared with controls: A Population-Based Study. *Arthritis & Rheumatology*.

[2] Shinomiya F., Mima N., Nanba K. (2008). Life expectancies of Japanese patients with rheumatoid arthritis: A review of deaths over a 20-year period. *Modern Rheumatology*.

[3] Nakajima A., Inoue E., Tanaka E. (2010). Mortality and cause of death in Japanese patients with rheumatoid arthritis based on a large observational cohort, IORRA. *Scandinavian Journal of Rheumatology*.

[4] Thomas C. F., Limper A. H. (2004). Pneumocystis pneumonia. *The New England Journal of Medicine*.

[5] Tokuda H., Sakai F., Yamada H. (2008). Clinical and radiological features of pneumocystis pneumonia in patients with rheumatoid arthritis, in comparison with methotrexate pneumonitis and pneumocystis pneumonia in acquired immunodeficiency syndrome: A multicenter study. *Internal Medicine*.

[6] Arnett F. C., Edworthy S. M., Bloch D. A. (1988). The American Rheumatism Association 1987 revised criteria for the classification of rheumatoid arthritis. *Arthritis & Rheumatism*.

[7] Aletaha D., Neogi T., Silman A. J. (2010). 2010 Rheumatoid arthritis classification criteria: an American College of Rheumatology/European League Against Rheumatism collaborative initiative. *Arthritis & Rheumatology*.

[8] Steinbrocker O., Traeger C. H., Batterman R. C. (1949). Therapeutic criteria in rheumatoid arthritis. *The Journal of the American Medical Association*.

[9] Hochberg M. C., Chang R. W., Dwosh I., Lindsey S., Pincus T., Wolfe F. (1992). The American College of Rheumatology 1991 revised criteria for the classification of global functional status in rheumatoid arthritis. *Arthritis & Rheumatism*.

[10] Matsuo S., Imai E., Horio M. (2009). Revised equations for estimated GFR from serum creatinine in Japan. *American Journal of Kidney Diseases*.

[11] Tasaka S., Hasegawa N., Kobayashi S. (2007). Serum indicators for the diagnosis of pneumocystis pneumonia. *CHEST*.

[12] Katsuyama T., Saito K., Kubo S., Nawata M., Tanaka Y. (2014). Prophylaxis for Pneumocystis pneumonia in patients with rheumatoid arthritis treated with biologics, based on risk factors found in a retrospective study. *Arthritis Research & Therapy*.

[13] Hashimoto A., Chiba N., Tsuno H. (2015). Incidence of malignancy and the risk of lymphoma in Japanese patients with rheumatoid arthritis compared to the general population. *The Journal of Rheumatology*.

[14] Smitten A. L., Choi H. K., Hochberg M. C. (2008). The risk of hospitalized infection in patients with rheumatoid arthritis. *The Journal of Rheumatology*.

[15] Au K., Reed G., Curtis J. R. (2011). High disease activity is associated with an increased risk of infection in patients with rheumatoid arthritis. *Annals of the Rheumatic Diseases*.

[16] Komano Y., Tanaka M., Nanki T. (2011). Incidence and risk factors for serious infection in patients with rheumatoid arthritis treated with tumor necrosis factor inhibitors: A report from the registry of Japanese rheumatoid arthritis patients for longterm safety. *The Journal of Rheumatology*.

[17] Crowson C. S., Hoganson D. D., Fitz-Gibbon P. D., Matteson E. L. (2012). Development and validation of a risk score for serious infection in patients with rheumatoid arthritis. *Arthritis & Rheumatology*.

[18] Sakai R., Komano Y., Tanaka M. (2012). Time-dependent increased risk for serious infection from continuous use of tumor necrosis factor antagonists over three years in patients with rheumatoid arthritis. *Arthritis Care & Research*.

[19] Ni Mhuircheartaigh O. M., Matteson E. L., Green A. B., Crowson C. S. (2013). Trends in serious infections in rheumatoid arthritis. *The Journal of Rheumatology*.

[20] Singh J. A., Cameron C., Noorbaloochi S. (2015). Risk of serious infection in biological treatment of patients with rheumatoid arthritis: a systematic review and meta-analysis. *The Lancet*.

[21] Widdifield J., Bernatsky S., Paterson J. M. (2013). Serious infections in a population-based cohort of 86,039 seniors with rheumatoid arthritis.. *Arthritis care & research*.

[22] Yun H., Xie F., Delzell E. (2016). Comparative Risk of Hospitalized Infection Associated with Biologic Agents in Rheumatoid Arthritis Patients Enrolled in Medicare. *Arthritis & Rheumatology*.

[23] Listing J., Gerhold K., Zink A. (2013). The risk of infections associated with rheumatoid arthritis, with its comorbidity and treatment. *Rheumatology*.

[24] Baum J. (1971). Infection in Rheumatoid Arthritis. *Arthritis & Rheumatism*.

[25] Kourbeti I. S., Ziakas P. D., Mylonakis E. (2014). Biologic therapies in rheumatoid arthritis and the risk of opportunistic infections: A meta-analysis. *Clinical Infectious Diseases*.

[26] Leombruno J. P., Einarson T. R., Keystone E. C. (2009). The safety of anti-tumour necrosis factor treatments in rheumatoid arthritis: meta and exposure-adjusted pooled analyses of serious adverse events. *Annals of the Rheumatic Diseases*.

[27] Greenberg J. D., Reed G., Kremer J. M. (2010). Association of methotrexate and tumour necrosis factor antagonists with risk of infectious outcomes including opportunistic infections in the CORRONA registry. *Annals of the Rheumatic Diseases*.

[28] Doran M. F., Crowson C. S., Pond G. R., O'Fallon W. M., Gabriel S. E. (2002). Predictors of infection in rheumatoid arthritis. *Arthritis & Rheumatology*.

[29] Sakai R., Komano Y., Tanaka M. (2011). The REAL database reveals no significant risk of serious infection during treatment with a methotrexate dose of more than 8 mg/week in patients with rheumatoid arthritis. *Modern Rheumatology*.

[30] Aaltonen K. J., Joensuu J. T., Virkki L. (2015). Rates of serious infections and malignancies among patients with rheumatoid arthritis receiving either tumor necrosis factor inhibitor or rituximab therapy. *The Journal of Rheumatology*.

[31] McLean-Tooke A., Aldridge C., Waugh S., Spickett G. P., Kay L. (2009). Methotrexate, rheumatoid arthritis and infection risk: what is the evidence?. *Rheumatology*.

[32] Iikuni N., Kitahama M., Ohta S., Okamoto H., Kamatani N., Nishinarita M. (2006). Evaluation of Pneumocystis pneumonia infection risk factors in patients with connective tissue disease. *Modern Rheumatology*.

[33] Tanaka M., Sakai R., Koike R. (2012). Pneumocystis jirovecii pneumonia associated with etanercept treatment in patients with rheumatoid arthritis: A retrospective review of 15 cases and analysis of risk factors. *Modern Rheumatology*.

[34] Komano Y., Harigai M., Koike R. (2009). Pneumocystis jiroveci pneumonia in patients with rheumatoid arthritis treated with infliximab: a retrospective review and case-control study of 21 patients. *Arthritis & Rheumatology*.

[35] Houtman P. M., Stenger A. A., Bruyn G. A., Mulder J. (1994). Methotrexate may affect certain T lymphocyte subsets in rheumatoid arthritis resulting in susceptibility to Pneumocystis carinii infection. *Journal of Rheumatology*.

[36] Li Y., Ghannoum M., Deng C. (2017). Pneumocystis pneumonia in patients with inflammatory or autoimmune diseases: Usefulness of lymphocyte subtyping. *International Journal of Infectious Diseases*.

[37] Mori S., Sugimoto M. (2012). Pneumocystis jirovecii infection: an emerging threat to patients with rheumatoid arthritis. *Rheumatology*.

[38] Mimori T., Harigai M., Atsumi T. (2016). Safety and effectiveness of 24-week treatment with iguratimod, a new oral disease-modifying antirheumatic drug, for patients with rheumatoid arthritis: interim analysis of a post-marketing surveillance study of 2679 patients in Japan. *Modern Rheumatology*.

[39] Louie G. H., Wang Z., Ward M. M. (2010). Trends in hospitalizations for Pneumocystis jiroveci pneumonia among patients with rheumatoid arthritis in the US: 1996-2007. *Arthritis & Rheumatology*.

[40] Bruce E. S., Kearsley-Fleet L., Watson K. D., etal. (2016). Risk of Pneumocystis jirovecii pneumonia in patients with rheumatoid arthritis treated with inhibitors of tumour necrosis factor *α*: results from the british society for rheumatology Biologics Register for Rheumatoid Arthritis. *Rheumatology*.

[41] Watanabe K., Sakai R., Koike R. (2013). Clinical characteristics and risk factors for Pneumocystis jirovecii pneumonia in patients with rheumatoid arthritis receiving adalimumab: A retrospective review and case-control study of 17 patients. *Modern Rheumatology*.

[42] Harigai M., Koike R., Miyasaka N. (2007). Pneumocystis pneumonia associated with infliximab in Japan. *The New England Journal of Medicine*.

[43] Mori S., Cho I., Ichiyasu H., Sugimoto M. (2008). Asymptomatic carriage of Pneumocystis jiroveci in elderly patients with rheumatoid arthritis in Japan: A possible association between colonization and development of Pneumocystis jiroveci pneumonia during low-dose MTX therapy. *Modern Rheumatology*.

[44] Fritzsche C., Riebold D., Munk-Hartig A. K., Klammt S., Neeck G., Reisinger E. C. (2012). High prevalence of Pneumocystis jirovecii colonization among patients with autoimmune inflammatory diseases and corticosteroid therapy. *Scandinavian Journal of Rheumatology*.

[45] Maskell N. A., Waine D. J., Lindley A. (2003). Asymptomatic carriage of Pneumocystis jiroveci in subjects undergoing bronchoscopy: A prospective study. *Thorax*.

[46] Wissmann G., Morilla R., Martín-Garrido I. (2011). Pneumocystis jirovecii colonization in patients treated with infliximab. *European Journal of Clinical Investigation*.

[47] Vidal S., de la Horra C., Martín J. (2006). *Pneumocystis jirovecii* colonisation in patients with interstitial lung disease. *Clinical Microbiology and Infection*.

[48] Calderón E. J., Rivero L., Respaldiza N. (2007). Systemic inflammation in patients with chronic obstructive pulmonary disease who are colonized with Pneumocystis jiroveci.. *Clinical infectious diseases : an official publication of the Infectious Diseases Society of America*.

[49] Mori S., Cho I., Sugimoto M. (2009). A followup study of asymptomatic carriers of Pneumocystis jiroveci during immunosuppressive therapy for rheumatoid arthritis. *The Journal of Rheumatology*.

